# 1-Aryl-3-(1*H*-imidazol-1-yl)propan-1-ol esters: synthesis, anti-*Candida* potential and molecular modeling studies

**DOI:** 10.1186/1752-153X-7-168

**Published:** 2013-10-25

**Authors:** Mohamed I Attia, Awwad A Radwan, Azza S Zakaria, Maha S Almutairi, Soraya W Ghoneim

**Affiliations:** 1 Department of Pharmaceutical Chemistry, College of Pharmacy, King Saud University, P.O. Box 2457, Riyadh 11451, Saudi Arabia; 2 Medicinal and Pharmaceutical Chemistry Department, Pharmaceutical and Drug Industries Research Division, National Research Centre, 12622, Dokki, Giza, Egypt; 3 Department of Pharmaceutics, College of Pharmacy, King Saud University, Riyadh, 11451, Saudi Arabia; 4 Department of Pharmaceutical Organic Chemistry, Faculty of Pharmacy, Assiut University, Assiut 71527, Egypt; 5 Department of Microbiology, Faculty of Pharmacy, Alexandria University, Alexandria, Egypt

**Keywords:** Synthesis, Mannich reaction, Imidazole, Esters, Anti-*Candida*, Molecular modeling

## Abstract

**Background:**

An increased incidence of fungal infections, both invasive and superficial, has been witnessed over the last two decades. *Candida* species seem to be the main etiology of nosocomial fungal infections worldwide with *Candida albicans*, which is commensal in healthy individuals, accounting for the majority of invasive *Candida* infections with about 30-40% of mortality.

**Results:**

New aromatic and heterocyclic esters **5a-k** of 1-aryl-3-(1*H*-imidazol-1-yl)propan-1-ols **4a-d** were successfully synthesized and evaluated for their anti-*Candida* potential. Compound **5a** emerged as the most active congener among the newly synthesized compounds **5a-k** with MIC value of 0.0833 μmol/mL as compared with fluconazole (MIC value >1.6325 μmol/mL). Additionally, molecular modeling studies were conducted on a set of anti-*Candida albicans* compounds.

**Conclusion:**

The newly synthesized esters **5a-k** showed more potent anti-*Candida* activities than fluconazole. Compounds **7** and **8** revealed significant anti-*Candida albicans* activity and were able to effectively satisfy the proposed pharmacophore geometry, using the energy accessible conformers (E_conf_ < 20 kcal/mol).

## Background

An increased incidence of fungal infections, both invasive and superficial, has been witnessed over the last two decades. Such infections are the major cause of morbidity and mortality especially in immune-compromised individuals such as patients with cancer or AIDS and in organ transplant cases [1,2]. *Candida* species seem to be the main etiology of nosocomial fungal infections worldwide with *Candida albicans*, which is commensal in healthy individuals [3], accounting for the majority of invasive *Candida* infections with about 30-40% of mortality [4]. Toxicity, low efficacy rates, and drug resistance limit the clinical use of the available antifungal agents [5]. This situation has led to an ongoing search to develop new potent broad spectrum antifungal agents with fewer side effects.

**Figure 1:**
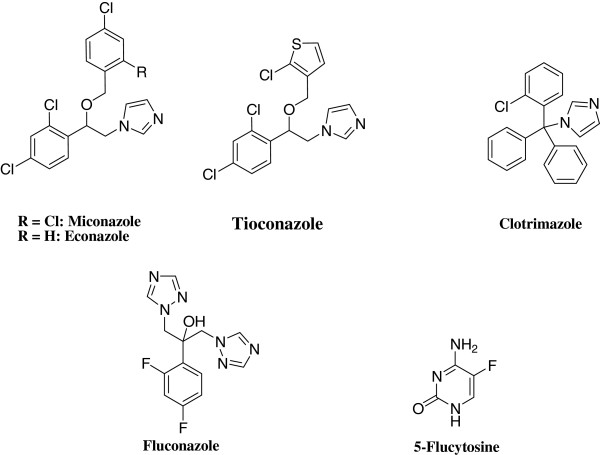
Chemical structures of common antifungal azole drugs.

The clinically used antifungal drugs belong to the classes of polyenes (such as amphotericin B and nystatin), echinocandins (such as caspofungin), allylamines (such as naftifine and terbinafine), fluoropyrimidines (such as 5-fluorocytosine) and azoles (such as miconazole and fluconazole) (Figure 1) [6-8]. Azole antifungal drugs featuring either an imidazole (e.g. miconazole, econazole, ketoconazole and clotrimazole) or a 1,2,4- triazole moiety (e.g. fluconazole and itraconazole) are the most widely used antifungal agents in clinics because of their safety profile and high therapeutic index [9]. The mechanism of action of azole antifungals relies on their ability to inhibit synthesis of sterols in fungi *via* inhibiting cytochrome P450-dependent 14α-lanosterol demethylase through binding to the heme cofactor of the cytochrome CYP51 [10,11].

An evaluation of the literature revealed that many imidazole-containing antifungal agents have a spacer of two carbon atoms between the imidazole pharmacophore and an aromatic moiety, but only limited information about imidazole-containing antifungals having a three-carbon atom bridge between the imidazole pharmacophore and the aromatic moiety is available [12,13]. Additionally, it has been well documented that some aryl and arylalkyl esters of 2-(1*H*-imidazol-1-yl)-1-phenylethanols displayed anti-*Candida albicans* activity more than that of miconazole [14].

Accordingly, we report herein the synthesis, anti-*Candida* activity and molecular modeling studies of certain new aryl/heterocyclic esters **5a-k** of 1-aryl-3-(1*H*-imidazol-1-yl)propan-1-ols **4a-d** bearing a three-carbon atom linker between the imidazole pharmacophore and the aromatic moiety.

### Experimental

Melting points were determined on a Gallenkamp melting point apparatus, and are uncorrected. NMR spectra were acquired on a Bruker NMR spectrometer operating at 500 MHz for ^1^H and 125.76 MHz for ^13^C at the Research Center, College of Pharmacy, King Saud University, Saudi Arabia. TMS was used as an internal standard and chemical shift values were recorded in ppm on the δ scale. The ^1^H NMR data were represented as follows: chemical shifts, multiplicity (s. singlet, d. doublet, t. triplet, m. multiplet, br. broad) and number of protons. The ^13^C NMR data were represented as chemical shifts and type of carbon. Mass spectra were measured on an Agilent Triple Quadrupole 6410 QQQ LC/MS with ESI (Electrospray ionization) source. Silica gel TLC (thin layer chromatography) plates from Merck (silica gel precoated aluminium plates with fluorescent indicator at 245 nm) were used for thin layer chromatography. Visualization was performed by illumination with a UV light source (254 nm). Column chromatography was carried out on silica gel 60 (0.063-0.200 mm) obtained from Merck and chloroform/methanol (18:1) was used as the solvent system. Our research group reported previously the synthesis and characterization of compounds **6–11**[12]. Fluconazole was obtained from Shouguang-Fukang Pharmaceutical Ltd., Shandong, China. The antifungal discs containing 25 μg fluconazole were purchased from ROSCO (Neo-Sensitabs, Taastrup, Denmark).

### General procedure for preparation of the ketones 3a-d

A mixture of the appropriate acetophenone **1a-d** (200 mmol), dimethylamine hydrochloride (260 mmol) and paraformaldehyde (260 mmol) in absolute ethanol (35 mL) and a catalytic amount of concentrated hydrochloric acid (0.5 mL). The reaction mixture was heated to reflux for two hours, cooled and acetone (200 mL) was added. The precipitated Mannich bases **2a-d** were filtered off and dried. Compounds **2a-d** (100 mmol) were dissolved in water (100 mL) and imidazole (200 mmol) was added. The reaction mixture was heated to reflux for five hours, cooled and the precipitated solids were collected by filtration to give the ketones **3a-d** which were pure enough to be used in the next step. The synthesized compounds **3a-d** gave the same analyses as reported previously [12,13,15,16].

### General procedure for preparation of the alcohols 4a-d

Sodium borohydride (130 mmol) was added portionwise to a solution of the appropriate ketone **3a-d** (43 mmol) in methanol (150 mL). The reaction mixture was stirred at ambient temperature for 18 hours. Methanol was evaporated under reduced pressure and the residue was dissolved in ethyl acetate (200 mL) and washed with water (3 × 50 mL). The organic phase was separated, dried (Na_2_SO_4_) and evaporated under vacuum to afford alcohols **4a-d** which were subjected to the subsequent esterification step without any further purification. The synthesized alcohols **4a-d** gave the same analyses as reported previously [12,13,17,18].

### General procedure for the synthesis of the target esters 5a-k

DMAP (400 mg) was added to a stirred solution of the appropriate acid (7 mmol) and EDCI HCl (7.3 mmol) in DCM (75 mL). The appropriate alcohol **4a-d** (6.9 mmol) was added to the stirred reaction mixture and stirring was continued for a further 18 h at room temperature. The reaction mixture was washed successively with water (2 × 20 mL), 10% NaHCO_3_ solution (2 × 15 mL), and water (2 × 15 mL). The organic layer was separated, dried (Na_2_SO_4_) and evaporated under reduced pressure and the residue was purified either by recrystallisation (for solids) or column chromatography (for oils).

### 3-(1*H*-Imidazol-1-yl)-1-phenylpropyl 3,4-dichlorobenzoate (5a)

Yield 65%; pale yellow viscous oil; IR (KBr): ν (cm^-1^) 3049, 2929, 1722 (C = O), 1505, 1266, 734; ^1^H NMR (CDCl_3_): δ 2.27-2.34 (m, 1H, -C*H*_2_-CH_2_-N), 2.47-2.54 (m, 1H, -C*H*_2_-CH_2_-N), 3.91-3.96 (m, 2H, -CH_2_-C*H*_*2*_-N), 5.85-5.88 (m, 1H, C_6_H_5_-C*H*-O-), 6.83 (s, 1H, -N-C*H* = CH-N=), 6.97 (s, 1H, -N-CH = C*H*-N=), 7.19-7.29 (m, 5H, Ar-H), 7.35 (s, 1H, -N-C*H* = N-), 7.34 (d, *J* = 8.4 Hz, 1H, Ar-H), 7.76, (dd, *J* = 8.4, 1.95 Hz, 1H, Ar-H_._), 7.99 (d, *J* = 1.93 Hz, 1H, Ar-H); ^13^C NMR (CDCl_3_): δ 36.4 (−*C*H_2_-CH_2_-N), 42.4 (−CH_2_-*C*H_2_-N), 73.5 (C_6_H_5_-*C*-O-), 117.7 (−N-*C*H = CH-N=), 125.3, 127.7, 127.8, 127.9, 128.6, 128.8, 129.7, 130.5, 132.0 (−N-CH = *C*H-N=, Ar-CH, Ar-C), 136.0 (−N-*C*H = N-), 136.9, 137.6 (Ar-C), 162.8 (C = O); MS m/z (ESI): Calcd for C_19_H_16_Cl_2_N_2_O_2_: 375.25. Found: 375.1 [M]^+^.

### 1-(4-Chlorophenyl)-3-(1*H*-imidazol-1-yl)propyl 4-chlorobenzoate (5b)

Yield 30%; pale yellow viscous oil; IR (KBr): ν (cm^-1^) 3052, 2985, 1717 (C = O), 1507, 1264, 738; ^1^H NMR (CDCl_3_): δ 2.33-2.39 (m, 1H, -C*H*_2_-CH_2_-N), 2.52-2.59 (m, 1H, -C*H*_2_-CH_2_-N), 4.09-4.13 (m, 2H, -CH_2_-C*H*_*2*_-N), 5.87-5.90 (m, 1H, C_6_H_5_-C*H*-O-), 6.92 (s, 1H, -N-C*H* = CH-N=), 7.09 (s, 1H, -N-CH = C*H*-N=), 7.19-7.29 (m, 5H, Ar-H, -N-C*H* = N-), 7.34-7.38 (m, 2H, Ar-H), 7.89-7.92 (m, 2H, Ar-H); ^13^C NMR (CDCl_3_): δ 37.4 (−*C*H_2_-CH_2_-N), 44.4 (−CH_2_-*C*H_2_-N), 73.2 (C_6_H_5_-*C*-O-), 119.4 (−N-*C*H = CH-N=), 127.5, 127.7, 128.7, 128.8, 129.0, 129.2, 131.1 (−N-CH = *C*H-N=, Ar-CH, Ar-C), 137.3 (−N-*C*H = N-), 139.5, 140.2 (Ar-C), 164.9 (C = O); MS m/z (ESI): Calcd for C_19_H_16_Cl_2_N_2_O_2_: 375.25. Found: 375.1 [M]^+^.

### 3-(1*H*-Imidazol-1-yl)-1-(4-methoxyphenyl)propyl 4-chlorobenzoate (5c)

Yield 28%; pale yellow viscous oil; IR (KBr): ν (cm^-1^) 3050, 2961, 1716 (C = O), 1513, 1265, 739; ^1^H NMR (CDCl_3_): δ 2.35-2.42 (m, 1H, -C*H*_2_-CH_2_-N), 2.58-2.65 (m, 1H, -C*H*_2_-CH_2_-N), 3.82 (s, 3H, OC*H*_3_), 4.01-4.08 (m, 2H, -CH_2_-C*H*_*2*_-N), 5.92-5.95 (m, 1H, C_6_H_5_-C*H*-O-), 6.93 (d, *J* = 9.0 Hz, 2H, Ar-H), 6.94 (s, 1H, -N-C*H* = CH-N=), 7.09 (s, 1H, -N-CH = C*H*-N=), 7.34 (d, *J* = 8.5 Hz, 2H, Ar-H), 7.43 (d, *J* = 8.5 Hz, 2H, Ar-H), 7.54 (s, 1H, -N-C*H* = N-), 7.97 (d, *J* = 8.5 Hz, 2H, Ar-H); ^13^C NMR (CDCl_3_): δ 37.4 (−*C*H_2_-CH_2_-N), 43.6 (−CH_2_-*C*H_2_-N), 55.3 (O*C*H_3_), 73.8 (C_6_H_5_-*C*-O-), 114.3 (Ar-CH), 118.8 (−N-*C*H = CH-N=), 127.9, 128.4, 128.8, 129.5, 130.8, 131.1 (−N-CH = *C*H-N=, Ar-CH, Ar-C), 137.0 (−N-*C*H = N-), 139.8, 159.9 (Ar-C), 164.8 (C = O); MS m/z (ESI): Calcd for C_20_H_19_ClN_2_O_3_: 370.83. Found: 371.2 [M + 1]^+^.

### 3-(1*H*-Imidazol-1-yl)-1-(4-methylphenyl)propyl 4-chlorobenzoate (5d)

Yield 36%; pale yellow viscous oil; IR (KBr): ν (cm^-1^) 3051, 2982, 1719 (C = O), 1506, 1264, 731; ^1^H NMR (CDCl_3_): δ 2.27 (s, 3H, C*H*_3_), 2.28-2.34 (m, 1H, -C*H*_2_-CH_2_-N), 2.47-2.54 (m, 1H, -C*H*_2_-CH_2_-N), 3.92-3.98 (m, 2H, -CH_2_-C*H*_*2*_-N), 5.84-5.87 (m, 1H, C_6_H_5_-C*H*-O-), 6.85 (s, 1H, -N-C*H* = CH-N=), 6.99 (s, 1H, -N-CH = C*H*-N=), 7.11 (d, *J* = 8.0 Hz, 2H, Ar-H), 7.19 (d, *J* = 8.0 Hz, 2H, Ar-H), 7.34 (d, *J* = 8.5 Hz, 2H, Ar-H), 7.45 (s, 1H, -N-C*H* = N-), 7.89 (d, *J* = 8.5 Hz, 2H, Ar-H); ^13^C NMR (CDCl_3_): δ 21.2 (*C*H_3_), 37.5 (−*C*H_2_-CH_2_-N), 43.6 (−CH_2_-*C*H_2_-N), 73.9 (C_6_H_5_-*C*-O-), 118.8 (−N-*C*H = CH-N=), 126.4, 128.3, 128.9, 129.4, 129.6, 131.1 (−N-CH = *C*H-N=, Ar-CH, Ar-C), 135.9, 137.0, 138.6, 139.8 (−N-*C*H = N-, Ar-C), 164.8 (C = O); MS m/z (ESI): Calcd for C_20_H_19_ClN_2_O_2_: 354.83. Found: 355.2 [M + 1]^+^.

### 3-(1*H*-Imidazol-1-yl)-1-phenylpropyl 4-fluorobenzoate (5e)

Yield 80%; pale yellow viscous oil; IR (KBr): ν (cm^-1^) 3051, 2979, 1721 (C = O), 1506, 1265, 738; ^1^H NMR (CDCl_3_): δ 2.27-2.34 (m, 1H, -C*H*_2_-CH_2_-N), 2.46-2.53 (m, 1H, -C*H*_2_-CH_2_-N), 3.91-3.99 (m, 2H, -CH_2_-C*H*_*2*_-N), 5.88-5.90 (m, 1H, C_6_H_5_-C*H*-O-), 6.84 (s, 1H, -N-C*H* = CH-N=), 6.98 (s, 1H, -N-CH = C*H*-N=), 7.04 (m, 2H, Ar-H), 7.23-7.29 (m, 5H, Ar-H), 7.40 (s, 1H, -N-C*H* = N-), 7.96-7.99, (m, 2H, Ar-H); ^13^C NMR (CDCl_3_): δ 37.7 (−*C*H_2_-CH_2_-N), 43.5 (−CH_2_-*C*H_2_-N), 73.9 (C_6_H_5_-*C*-O-), 115.7 (d, *J* = 22.0 Hz, Ar-CH), 118.8 (−N-*C*H = CH-N=), 126.1, (d, *J* = 2.4 Hz, Ar-C), 126.3 (Ar-CH), 128.7, 128.9 (−N-CH = *C*H-N=, Ar-CH), 129.7 (Ar-CH), 132.3 (d, *J* = 9.4 Hz, Ar-CH), 137.1 (−N-*C*H = N-), 139.0 (Ar-C), 164.7 (C = O), 165.8 (d, *J* = 254.7 Hz, Ar-C); MS m/z (ESI): Calcd for C_19_H_17_FN_2_O_2_: 324.35. Found: 325.2 [M + 1]^+^.

### 3-(1*H*-Imidazol-1-yl)-1-phenylpropyl 4-methylbenzoate (5f)

Yield 52%; pale yellow solid, m.p. 60–62°C (isopropanol); IR (KBr): ν (cm^-1^) 3049, 2976, 1717 (C = O), 1506, 1266, 739; ^1^H NMR (CDCl_3_): δ 2.27-2.32 (m, 1H, -C*H*_2_-CH_2_-N), 2.34 (s, 3H, C*H*_*3*_), 2.45-2.52 (m, 1H, -C*H*_2_-CH_2_-N), 3.92-4.01 (m, 2H, -CH_2_-C*H*_*2*_-N), 5.89-5.92 (m, 1H, C_6_H_5_-C*H*-O-), 6.85 (s, 1H, -N-C*H* = CH-N=), 6.99 (s, 1H, -N-CH = C*H*-N=), 7.18 (d, *J* = 7.8 Hz, 2H, Ar-H), 7.23-7.31 (m, 5H, Ar-H), 7.42 (s, 1H, -N-C*H* = N-), 7.87, (d, *J* = 8.1 Hz, 2H, Ar-H); ^13^C NMR (CDCl_3_): δ 21.7 (*C*H_3_), 37.9 (−*C*H_2_-CH_2_-N), 43.5 (−CH_2_-*C*H_2_-N), 73.4 (C_6_H_5_-*C*-O-), 118.8 (−N-*C*H = CH-N=), 126.3, 127.1, 128.5, 128.9, 129.3, 129.5, 129.7 (−N-CH = *C*H-N=, Ar-CH, Ar-C), 137.0 (−N-*C*H = N-), 139.4, 144.2 (Ar-C), 165.7 (C = O); MS m/z (ESI): Calcd for C_20_H_20_N_2_O_2_: 320.38. Found: 321.2 [M + 1]^+^.

### 3-(1*H*-Imidazol-1-yl)-1-phenylpropyl 4-(trifluoromethyl)benzoate (5g)

Yield 49%; white solid, m.p. 98–100°C (isopropanol); IR (KBr): ν (cm^-1^) 3053, 2979, 1717 (C = O), 1506, 1269, 736; ^1^H NMR (CDCl_3_): δ 2.29-2.36 (m, 1H, -C*H*_2_-CH_2_-N), 2.49-2.56 (m, 1H, -C*H*_2_-CH_2_-N), 3.91-3.99 (m, 2H, -CH_2_-C*H*_*2*_-N), 5.89-5.92 (m, 1H, C_6_H_5_-C*H*-O-), 6.84 (s, 1H, -N-C*H* = CH-N=), 6.98 (s, 1H, -N-CH = C*H*-N=), 7.23-7.31 (m, 5H, Ar-H), 7.39 (s, 1H, -N-C*H* = N-), 7.62 (d, *J* = 8.2 Hz, 2H, Ar-H), 8.06, (d, *J* = 8.5 Hz, 2H, Ar-H); ^13^C NMR (CDCl_3_): δ 37.5 (−*C*H_2_-CH_2_-N), 43.4 (−CH_2_-*C*H_2_-N), 74.5 (C_6_H_5_-*C*-O-), 118.7 (−N-*C*H = CH-N=), 125.6 (d, *J* = 3.4 Hz, *C*F_3_), 126.4, 128.8, 128.9, 129.7, 130.1, 133.0 (−N-CH = *C*H-N=, Ar-CH,), 134.7, 134.9, 137.0, 138.7 (−N-*C*H = N-, Ar-C), 164.4 (C = O); MS m/z (ESI): Calcd for C_20_H_17_F_3_N_2_O_2_: 374.36. Found: 375.2 [M + 1]^+^.

### 3-(1*H*-Imidazol-1-yl)-1-phenylpropyl furan-2-carboxylate (5h)

Yield 68%; white solid, m.p. 102–104°C (isopropanol); IR (KBr): ν (cm^-1^) 3121, 2921, 1716 (C = O), 1511, 1299, 775; ^1^H NMR (CDCl_3_): δ 2.25-2.32 (m, 1H, -C*H*_2_-CH_2_-N), 2.44-2.52 (m, 1H, -C*H*_2_-CH_2_-N), 3.92-4.01 (m, 2H, -CH_2_-C*H*_*2*_-N), 5.87-5.89 (m, 1H, C_6_H_5_-C*H*-O-), 6.44 (dd, *J* = 1.7, 3.5 Hz, 1H, furan-H), 6.85 (s, 1H, -N-C*H* = CH-N=), 6.99 (s, 1H, -N-CH = C*H*-N=), 7.14 (dd, *J* = 0.5, 3.5 Hz, 1H, furan-H), 7.22-7.29 (m, 5H, Ar-H), 7.43 (s, 1H, -N-C*H* = N-), 7.51-7.52, (m, 1H, furan-H); ^13^C NMR (CDCl_3_): δ 37.7 (−*C*H_2_-CH_2_-N), 43.4 (−CH_2_-*C*H_2_-N), 73.6 (C_6_H_5_-*C*-O-), 112.0 (furan-CH), 118.6 (furan-CH), 118.8 (−N-*C*H = CH-N=), 126.4, 128.7, 128.9, 129.5 (−N-CH = *C*H-N=, Ar-CH), 137.0 (−N-*C*H = N-), 138.4 (Ar-C), 144.3 furan-C), 146.7 (furan-CH), 157.7 (C = O); MS m/z (ESI): Calcd for C_17_H_16_N_2_O_3_: 296.32. Found: 297.2 [M + 1]^+^.

### 3-(1*H*-Imidazol-1-yl)-1-phenylpropyl pyridine-3-carboxylate (5i)

Yield 86%; off white solid, m.p. 96–98°C (isopropanol); IR (KBr): ν (cm^-1^) 3059, 2979, 1724 (C = O), 1506, 1265, 733; ^1^H NMR (CDCl_3_): δ 2.39-2.47 (m, 1H, -C*H*_2_-CH_2_-N), 2.60-2.68 (m, 1H, -C*H*_2_-CH_2_-N), 4.02-4.11 (m, 2H, -CH_2_-C*H*_*2*_-N), 6.00-6.03 (m, 1H, C_6_H_5_-C*H*-O-), 6.95 (s, 1H, -N-C*H* = CH-N=), 7.09 (s, 1H, -N-CH = C*H*-N=), 7.35-7.41 (m, 6H, Ar-H, Pyridine-H5), 8.81 (dd, *J* = 1.5, 5.0 Hz, 1H, Pyridine-H6), 7.54 (s, 1H, -N-C*H* = N-), 8.28-8.29 (m, 1H, Pyridine-H4), 9.26 (d, *J* = 1.5 Hz, 1H, Pyridine-H2); ^13^C NMR (CDCl_3_): δ 37.5 (−*C*H_2_-CH_2_-N), 43.5 (−CH_2_-*C*H_2_-N), 74.4 (C_6_H_5_-*C*-O-), 118.7 (−N-*C*H = CH-N=), 123.4 (pyridine-C5), 125.8, 126.4, 128.8, 128.9, 129.6 (−N-CH = *C*H-N=, Ar-CH, pyridine-C3), 137.0, 138.6 (−N-*C*H = N-, Ar-C), 150.9 (pyridine-C2), 153.8 (pyridine-C6), 164.4 (C = O); MS m/z (ESI): Calcd for C_18_H_17_N_3_O_2_: 307.35. Found: 308.2 [M + 1]^+^.

### 3-(1*H*-Imidazol-1-yl)-1-phenylpropyl pyridine-4-carboxylate (5j)

Yield 60%; pale yellow viscous oil; IR (KBr): ν (cm^-1^) 3051, 2981, 1730 (C = O), 1506, 1264, 737; ^1^H NMR (CDCl_3_): δ 2.32-2.39 (m, 1H, -C*H*_2_-CH_2_-N), 2.52-2.59 (m, 1H, -C*H*_2_-CH_2_-N), 3.93-4.02 (m, 2H, -CH_2_-C*H*_*2*_-N), 5.89-5.92 (m, 1H, C_6_H_5_-C*H*-O-), 6.85 (s, 1H, -N-C*H* = CH-N=), 6.99 (s, 1H, -N-CH = C*H*-N=), 7.25-7.34 (m, 5H, Ar-H), 7.46 (s, 1H, -N-C*H* = N-), 7.76 (d, *J* = 6.0 Hz, 2H, Pyridine-H3, H5), 8.72 (d, *J* = 6.0 Hz, 2H, Pyridine-H2, H6); ^13^C NMR (CDCl_3_): δ 37.4 (−*C*H_2_-CH_2_-N), 43.5 (−CH_2_-*C*H_2_-N), 74.8 (C_6_H_5_-*C*-O-), 118.7 (−N-*C*H = CH-N=), 122.8 (pyridine-C3, C5), 126.4, 128.9, 128.9, 129.0 (−N-CH = *C*H-N=, Ar-CH), 137.0, 138.4 (−N-*C*H = N-, Ar-C), 150.8 (pyridine-C2, C6), 164.2 (C = O); MS m/z (ESI): Calcd for C_18_H_17_N_3_O_2_: 307.35. Found: 308.2 [M + 1]^+^.

### 3-(1*H*-Imidazol-1-yl)-1-phenylpropyl 5-chlorothiophene-2-carboxylate (5k)

Yield 80%; white solid, m.p. 96–98°C (isopropanol); IR (KBr): ν (cm^-1^) 3050, 2981, 1717 (C = O), 1506, 1265, 736; ^1^H NMR (CDCl_3_): δ 2.34-2.41 (m, 1H, -C*H*_2_-CH_2_-N), 2.52-2.59 (m, 1H, -C*H*_2_-CH_2_-N), 4.00-4.09 (m, 2H, -CH_2_-C*H*_*2*_-N), 5.90-5.93 (m, 1H, C_6_H_5_-C*H*-O-), 6.94 (s, 1H, -N-C*H* = CH-N=), 6.97 (d, *J* = 4.0 Hz, 1H, thiophene-H), 7.09 (s, 1H, -N-CH = C*H*-N=), 7.35-7.41 (m, 5H, Ar-H), 7.52 (s, 1H, -N-C*H* = N-), 7.63 (d, *J* = 4.0 Hz, 1H, thiophene-H); ^13^C NMR (CDCl_3_): δ 37.7 (−*C*H_2_-CH_2_-N), 43.4 (−CH_2_-*C*H_2_-N), 74.2 (C_6_H_5_-*C*-O-), 118.7 (−N-*C*H = CH-N=), 126.3, 127.5, 128.7, 128.9, 129.6 (−N-CH = *C*H-N=, thiophene-CH, Ar-CH), 131.3 (thiophene-CH), 133.6 (thiophene-C), 137.0 (−N-*C*H = N-), 137.9 (thiophene-C), 138.7 (Ar-C), 160.2 (C = O); MS m/z (ESI): Calcd for C_17_H_15_ClN_32_O_2_S: 346.83. Found: 347.2 [M + 1]^+^.

### Anti-*Candida* activity

#### Anti-Candida agents

Stock solutions (1000 μg/mL) of fluconazole and/or the synthesized compounds **5a-k** and **6–11** were prepared in 100% dimethyl sulfoxide and were diluted with sterile distilled water. All antifungal discs were stored at −80°C until used.

#### Media

Liquid RPMI 1640 medium supplemented with L-glutamine was purchased from Sigma-Aldrich Co. (St. Louis, MO, USA) and was added to 2% sodium bicarbonate and 0.165 M morpholinepropane sulfonic acid (MOPS) from Dojindo Laboratories (Kumamoto, Japan) then adjusted to pH 7.0. Sabouraud Dextrose Agar (SDA) and Brain Heart Infusion (BHI) were purchased from Difco Laboratories (Detroit. Michigan, USA). Potato Dextrose Agar (PDA) was purchased from Eiken Chemical Co. Ltd. (Tokyo, Japan).

#### Organisms

Two clinical isolates of *Candida* species, one identified as *C*. *albicans* and the other as *C. tropicalis*, were obtained from King Khaled Hospital, Riyadh, Saudi Arabia*.* The yeasts were stored at −70°C in BHI with glycerol 5% until tested.

#### Preparation of inocula

Preparation of inocula for the broth microdilution testing was performed in accordance with CLSI documents M27-A2 [19] with RPMI 1640 medium. Isolates of *Candida* species were subcultured at 35°C for 48 h on PDA plates. Yeast cells were recovered from at least five 1-mm-diameter colonies and suspended in 5 mL of sterile saline. The suspension was mixed for 15 s with a vortex mixer, and the turbidity of each suspension was adjusted to a 0.5 McFarland standard (corresponding to 1.3 ×10^6^ to 5.3 × 10^6^ CFU/mL) at a wavelength of 530 nm according to the reported method [19]. Each suspension was diluted 1,000-fold with RPMI 1640 medium to give a final inoculum of 1.3 × 10^3^ to 5.3 × 10^3^ CFU/mL.

#### Disk diffusion assay

The disk diffusion assay was performed as described previously [20]. Cell suspensions of the previously chosen yeasts were adjusted to a 0.5 McFarland standard (corresponding to 5 × 10^6^ CFU/mL). A 100 μl suspension of each tested strain was spread uniformly onto SDA plates. Whatmann filter paper disks with a diameter of 6 mm were impregnated with 1000 μg of the synthesized compounds **5a-k**. After the disks were allowed to dry, they were placed onto the surface of the inoculated agar plates together with the standard antifungal discs which were then incubated at 35°C. Experiments were performed in duplicate and diameters of inhibition zones were measured at 24 hours.

#### Antifungal susceptibility studies

The previously prepared yeast inocula (100 μl) were added to each well of 96-well flat-bottom microdilution plates; each well contained 100 μl of twofold serial dilutions of the reference standard and/or the synthesized compounds **5a-k** and **6–11** ranging from 1 μg/mL to 500 μg/mL in RPMI 1640 medium. After each plate was incubated at 35°C for 48 h, the turbidity of each well was measured at 490 nm with a microplate ELISA reader. The MICs of the *Candida* species were recorded as the lowest concentration at which a prominent decrease (50-80%) in turbidity relative to the turbidity of the growth control was observed.

### Pharmacophore modeling

LigandScout (version 3.0) was used to derive the 3D chemical feature-based pharmacophores from the structural data of the compounds under study **5a-k** and **6–11**, using the default settings [21]. Compounds **5a**, **5d**, **5j**, **6–8**, **10** and **11** were included as a training set in the modeling study. Prior to the generation of pharmacophore hypotheses, the training set compounds, which were converted to 3D structure, were used to generate diverse conformations. The LigandScout program was used to generate the conformations, using the BEST conformation model generation method. Other parameters, such as the maximum number of 250 conformers and an energy threshold value of 20 kcal/mol above the local energy minimum, were chosen during conformations generation. The pharmacophoric features, hydrogen bond acceptor (HBA), hydrogen bond donor (HBD) and hydrophobic feature (HY), were selected to produce a reliable pharmacophore model for the experimental results during the pharmacophore hypothesis generation. Compounds **5b**, **5c**, **5e-i**, **5k** and **9**, were selected as a test set for pharmacophore validation. This method is used to elucidate whether the generated pharmacophore hypothesis is proficient to predict the activities of compounds other than the training set and classify them correctly in their activity scale. The conformation generation for the test set compounds was carried out in a similar way, as the training set compounds using the BEST conformational analysis algorithm, implemented within LigandScout. Subsequently, pharmacophore mapping was conducted on the resulting conformations [21].

### Docking procedure

All molecular modeling studies were performed on a PC using Windows Vista Home Premium Intel(R) Core(TM)2 Duo, 1.83GHz using Dock6.4 [22]. All compounds were generated in the protonated state that would be found under physiological conditions. The coordinates of the X-ray structure of 14-α-sterol demethylase (CYP51) active site domain complexed with fluconazol (1EA1) was obtained from the Protein Data Bank [23]. The co-crystallized ligand was docked in its original protein structure using the default settings of the program. A 10 Å sphere around the centre of the active site was defined as the binding pocket for the docking runs. All torsion angles in each compound were allowed to rotate freely.

## Results and discussion

### Chemistry

**Scheme 1:**
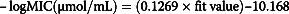
**Synthesis of the ketones 3a-d.** Reagents and conditions: i) HN(CH_3_)_2_HCl, (CH_2_O)_n_, conc. HCl, ethanol, reflux, 2 h; 47-95% yield ii) Imidazole, water, reflux, 5 h, 44-77% yield.

**Scheme 2:**
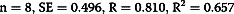
**Synthesis of the target compounds 5a-k.** Reagents and conditions: i) NaBH_4_, methanol, rt, 24 h; 60-78% yield ii) Appropriate carboxylic acid, EDCI HCl, DMAP, DCM, rt, 18 h, 28-86% yield.

The synthetic strategy to prepare the target compounds **5a-k** and their intermediates is illustrated in Schemes 1 and 2. Preparation of the pivotal ketones **3a-d** began by allowing the appropriate acetophenone **1a-d** to react with dimethylamine hydrochloride and paraformaldehyde in the presence of a catalytic amount of concentrated hydrochloric acid. The formed Mannich base hydrochlorides **2a-d** were transformed into the ketones **3a-d***vi*a refluxing with imidazole in water (Scheme 1).

Ketones **3a-d** were reduced to their respective alcohols **4a-d** using sodium borohydride in methanol. The resulting alcohols **4a-d** were subjected to esterification with the appropriate carboxylic acid derivatives using ethyl-3-(3-dimethylaminopropyl)carbodiimide hydrochloride (EDCI HCl) in the presence of 4-dimethylaminopyridine (DMAP) to yield the target compounds **5a-k** (Scheme 2). The chemical structures of the title compounds **5a-k** were confirmed *via* IR, ^1^H NMR, ^13^C NMR and mass spectral data.

### *In vitro* anti-*Candida* activity

The gold standard azole antifungal, fluconazole, has been used as a first line of treatment for fungal infections especially those caused by *C. albicans*, but its extensive use in the clinics has led to the development of resistance [24]. The *in vitro* anti-*Candida* activity of the synthesized imidazole-containing esters **5a-k** was evaluated against two clinical isolates of *Candida*: *C*. *albicans* and *C. tropicalis,* which were practically resistant to fluconazole (MIC >1.6325 μmol/mL). The obtained data, expressed as diameter of the inhibition zone (DIZ) and minimum inhibition concentration (MIC) for the test compounds **5a-k** and for the reference drug, fluconazole, are presented in Table 1.

**Table 1:** **Anti-*Candida *** **activity of the target compounds 5a-k against *Candida albicans *** **and *Candida tropicalis***

**Compound no**	***Candida albicans***		***Candida tropicalis***	
	DIZ ± SD*	MIC (μmol/mL)**	DIZ ± SD*	MIC (μmol/mL)**
**5a**	17 ± 1.1	0.0833	13 ± 0.9	0.3331
**5b**	16 ±1.1	0.6662	14 ±1.0	0.3331
**5c**	16 ±1.0	0.6742	14 ± 0.9	0.3371
**5d**	14 ± 0.5	0.3523	16 ±1.1	0.3523
**5e**	16 ±1.6	0.3854	16 ± 0.6	0.3854
**5f**	16 ± 0.9	0.3902	11 ± 0.9	0.7803
**5g**	14 ± 0.0	0.6678	14 ± 0.0	0.3339
**5h**	17 ± 0.9	0.4218	17 ± 0.8	0.4218
**5i**	14 ± 0.0	0.8134	16 ± 1.0	0.8134
**5j**	14 ± 0.0	0.8134	19 ± 1.0	0.4067
**5k**	16 ± 0.6	0.7208	14 ± 0.5	0.3604
Fluconazole	15 ± 0.5	>1.6325	16 ± 0.5	>1.6325

*The arithmetic mean of the inhibition zone diameters in mean ± standard deviation. **The lowest concentration of the compound that produced 50-80% microbial growth inhibition (μmol/mL).

**Figure 2:**
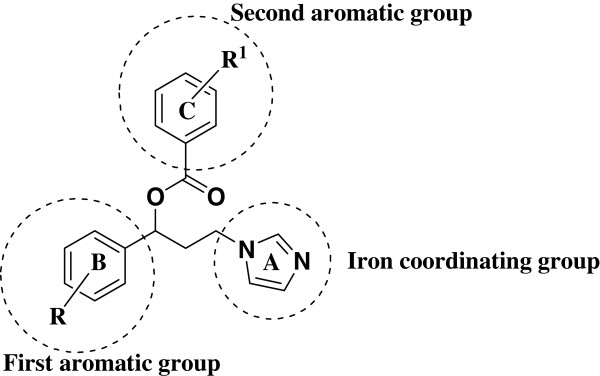
Schematic representation of the pharmacophore of azole antifungal aromatic esters.

It was previously reported that the alcohol **4a** displayed a weak antifungal activity [12] which is consistent with the proposed pharmacophore model of azole antifungals (Figure 2) [25]. This azole antifungal pharmacophore model incorporates at least three binding points, namely iron coordinating group, first aromatic group and a second aromatic group, to elicit optimum activity. Alcohol **4a** possesses only two binding points, imidazole moiety and an aromatic moiety B adjacent to it. Accordingly, addition of a third binding point *via* aromatic esterification of the alcohol functionality of **4a** should enhance its antifungal activity.

The preliminary anti-*Candida* potential of the test compounds **5a-k** was evaluated using DIZ assay and the results are illustrated in Table 1. Compounds **5a-k** showed a promising anti-*Candida* profile with DIZ = 14–17 mm and 11–19 mm towards *C*. *albicans* and *C*. *tropicalis*, respectively. Compounds **5a** and **5h** were the most active congeners against *C*. *albicans* while compound **5j** was the most active candidate against *C*. *tropicalis*. Consequently, MIC values of the test compounds **5a-k** were determined in order to gain insight into their anti-*Candida* activity as compared with that of fluconazole, the gold standard antifungal drug.

The synthesized aromatic esters of **4a**, compounds **5a** and **5e-g**, showed better anti-*Candida* activity (MICs = 0.0833-0.6678 and 0.3331-0.7803 μmol/mL towards *C. albicans* and *C. tropicalis*, respectively) than that of fluconazole (MIC >1.6325 μmol/mL). Additionally, esterification of the alcohol functionality of **4a** with aromatic isoster heterocyclic carboxylic acids gave compounds **5h-k**, which exhibited good anti-*Candida* activity (MICs = 0.4218-0.8134 and 0.3604-0.8134 μmol/mL towards *C. albicans* and *C. tropicalis*, respecively) being more potent than fluconazole (MIC >1.6325 μmol/mL).

**Figure 3:**
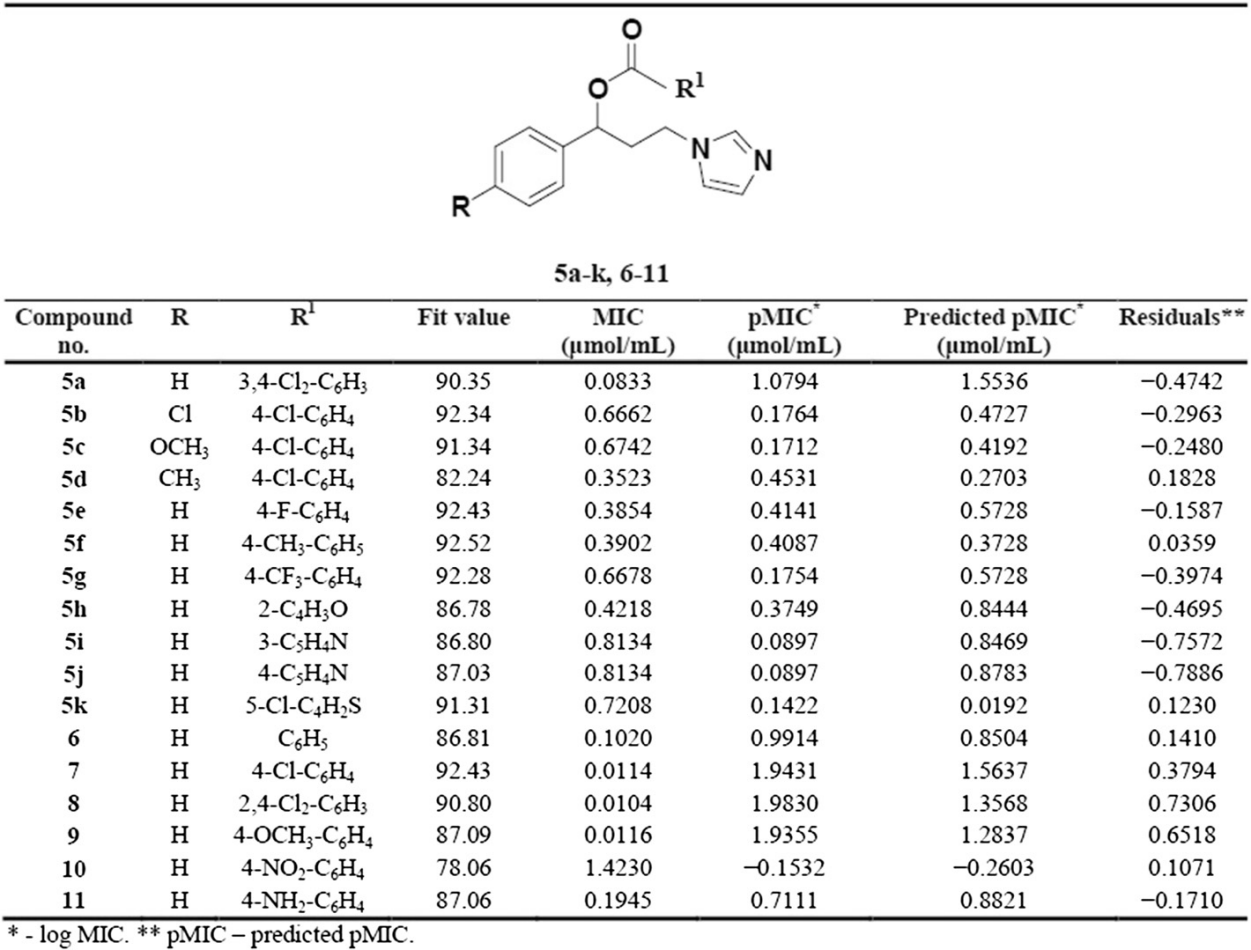
**The pharmacophore model mapping and predictive values of compounds 5a-k and 6-11 as anti-*Candida albicans *** **agents.**

It was previously documented that esterification of **4a** with 4-chlorobenzoic acid gave compound **7** (Figure 3) which showed potent anti-*Candida* activity [12]. Substitution of aromatic ring B of compound **7** with chloro, methoxy and/or methyl groups gave compounds **5b**, **5c** and **5d**, respectively. Compounds **5b-d** exhibited anti-*Candida albicans* activity (MICs = 0.3523-0.6742 μmol/mL) weaker than that of their parent compound **7** (MIC = 0.0114 μmol/mL) but they are more potent than fluconazole (MIC >1.6325 μmol/mL) (Figure 3).

### Pharmacophore modeling

**Figure 4:**
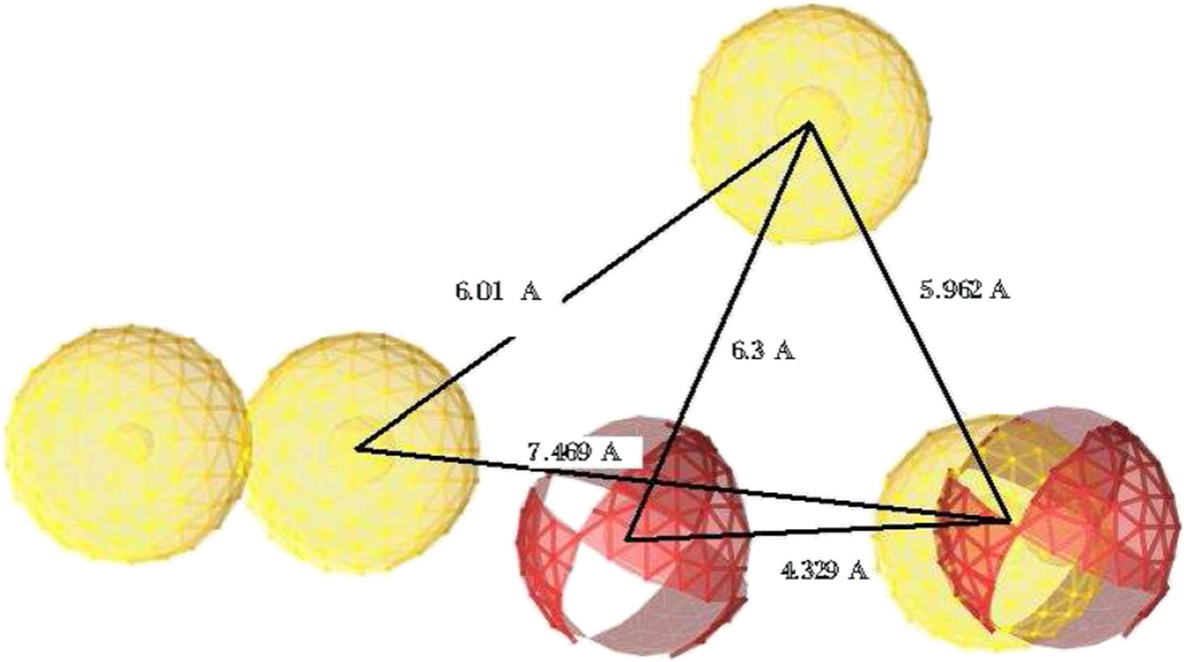
**Proposed pharmacophore model of growth inhibition activity against *Candida albicans *** **(red, HBA; yellow, hydrophobic).**

In view of the excellent *in vitro* results, additional *in silico* studies were done to support the *in vitro* anti-*Candida* studies. The anti-*Candida* potential of the synthesized compounds **5a-k** and **6–11** (Figure 3), against *Candida albicans*, was selected for a pharmacophore modeling study. Compounds **5a**, **5d**, **5j**, **6–8**, **10** and **11** were used as a training set while compounds **5b**, **5c**, **5e-i**, **5k** and **9** were used as a test set. On the assumption that the active compounds bind in a similar fashion to the active site, we employed the LigandScout program [26] to evaluate common features essential for activity, and the hypothetical geometries adopted by these ligands in their most active forms. Thus, these compounds were submitted for pharmacophore model generation based on shared chemical features. Conformations within 20 kcal/mol were submitted to the alignment procedure. The successful pharmacophore run resulted in generation of 10 models. Based on its highest score and its mapping into all training set molecules, the chosen model was statistically considered to be the best hypothesis and was selected for further investigation and analysis. The proposed pharmacophore model is shown in Figure 4 and contains six chemical features: four hydrophobic regions and two hydrogen bond acceptors.

**Figure 5:**
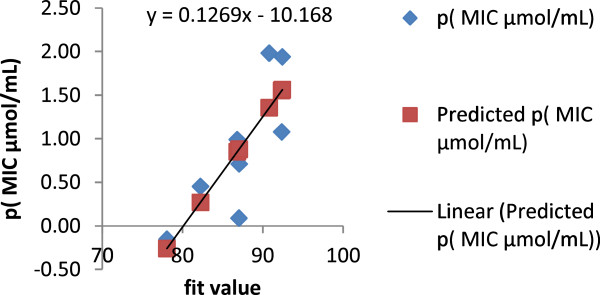
A chart representing the negative logarithm of the minimal inhibitor concentration in μmol/mL (dependent value) against LigandScout program output fit value (independent value).

All of the synthesized compounds, **5a-k** and **6–11**, were mapped onto the model, and the orientation of the mapped compound relative to the proposed pharmacophore was scored ("fit value") (Figure 3). This provided a quick validation of the model with mapped compounds showing good agreement with the fit value and biological activity (Figure 3). The highly active compounds showed a range of fit values of 90.8–92.43, while the remaining compounds with moderate to weak activity, showing fit values of 78.06–87.09. This initial correlation encouraged us to generate a linear model, based on “fit value” to predict the biological activity of the test set compounds. The generated model (Equation 1) was used successfully to predict the activity of the test set compounds (Figures 3 and 5).

(1) $$-\log\mathrm{MIC}\left(µ\text{mol}/\mathrm{mL}\right)=\left(0.1269\times \mathrm{fit}\;\mathrm{value}\right)-10.168$$

$$n=8\text{,}\;\text{SE}=0.496\text{,}\;R=0.810\text{,}\;R^{2}=0.657$$

Where n: number of compounds; R: multiple correlation coefficients; SE: standard error.

**Figure 6:**
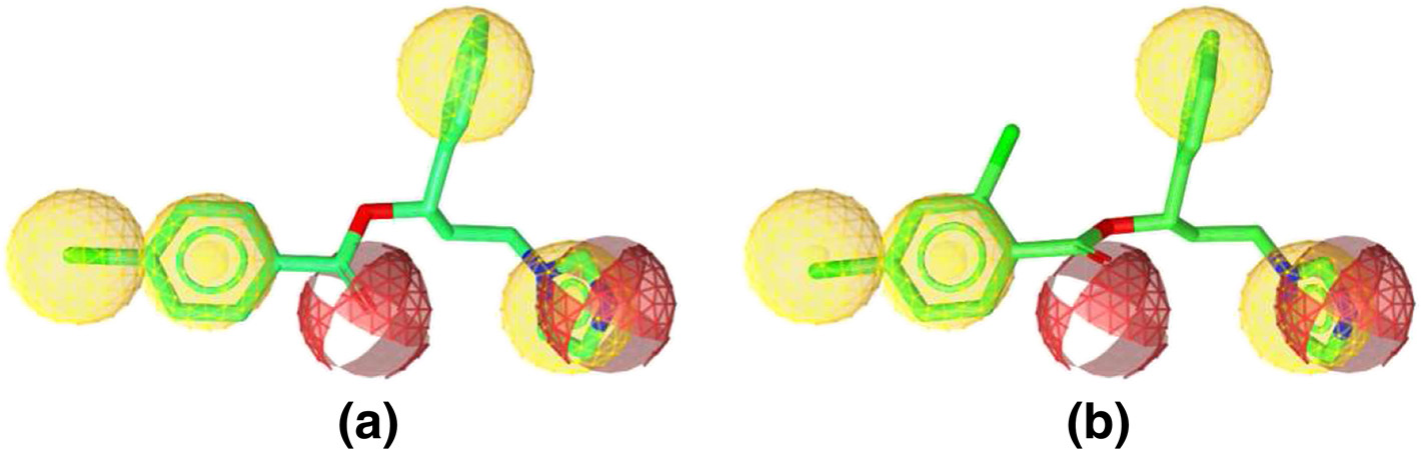
**Aligned poses of compounds 7 and 8. (a)** Best aligned pose of compound **7** (MIC 0.0114 μmol/mL) superposed with the query (model-1). **(b)** Best aligned pose of compound **8** (MIC 0.0104 μmol/mL) fitted inadequately with the query (model-1).

**Figure 7:**
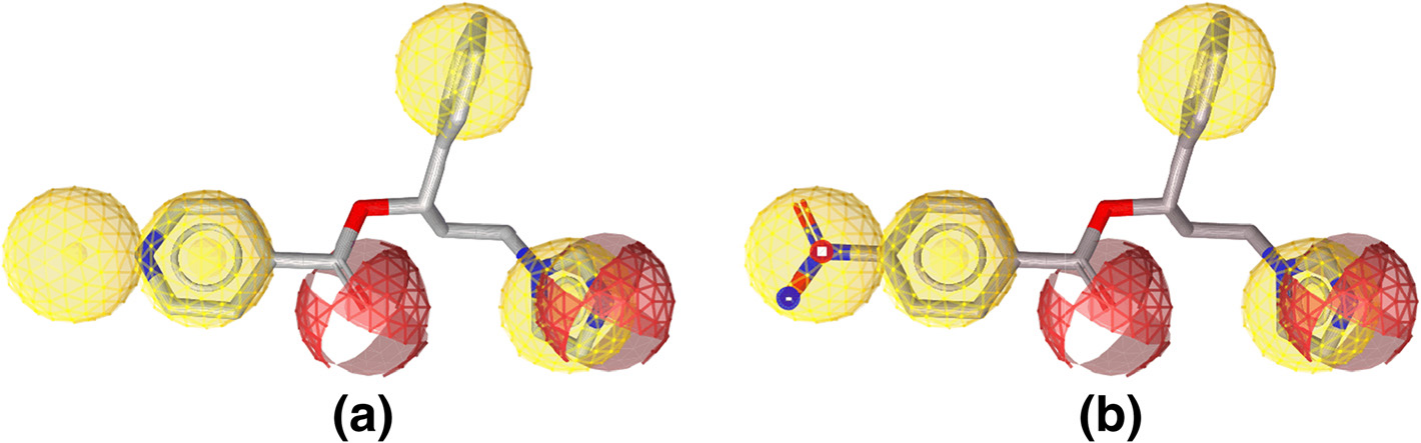
**Aligned poses of compounds 5j and 10. (a)** Best aligned pose of the less active compound **5j** (MIC 0.8134 μmol/mL) overlaid onto the pharmacophore model (model-1). **(b)** Best aligned pose of the less active compound **10** (MIC 1.4230 μmol/mL) overlaid onto the pharmacophore model (model-1).

Figure 6 shows the alignment of the hypothesis model with the most active compounds **7** and **8**, and Figure 7 shows the alignment of the hypothesis model with the weakly active compounds **5j** and **10**. A closer look at the mapped structures (Figure 6) reveals the importance of certain structural features for activity. The presence of a hydrophobic substituent at the para position of the benzoate moiety appears to be preferable for the anti-*Candida albicans* activity of the tested compounds. Unsubstituted benzoate or para-substituted with polar group (Figure 7) affects badly on the fit value of the compounds to the pharmacophore model, which is also reflected on its experimental weak activity.

### Docking procedure

**Figure 8:**
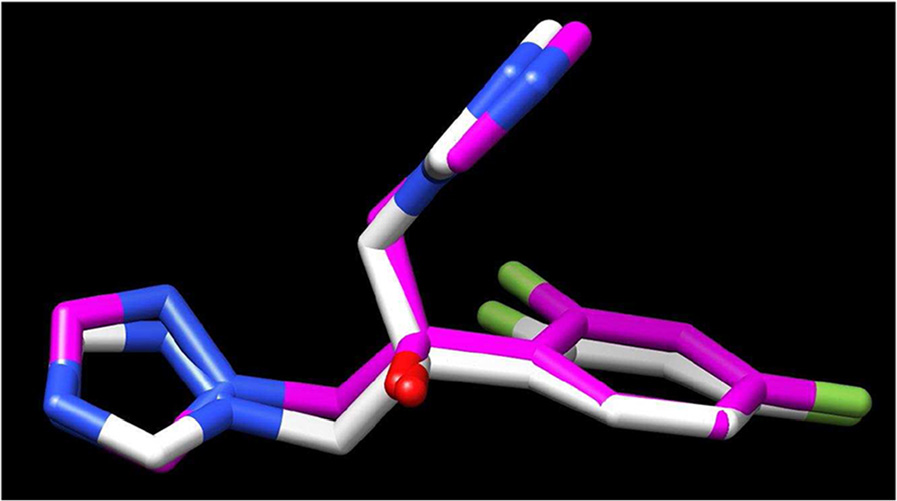
Superimposition of the co-crystallized FCZ (from 1AE1.pdb, colored magenta) and the re-docked FCZ (colored white).

A docking study was undertaken using Dock6.4 [22] in order to investigate the possible interactions between the designed compounds and the active site of the CYP51 enzyme. The X-ray structure of the enzyme bound with fluconazole (FCZ) was taken from the protein data bank; PDB code: 1EA1 [23]. The RMSD value difference of 0.431 Å of the pose of the non-restricted redocking of FCZ into the X-ray structure of the CYP51 from the co-crystallized FCZ also confirmed the approach (Figure 8).

**Figure 9:**
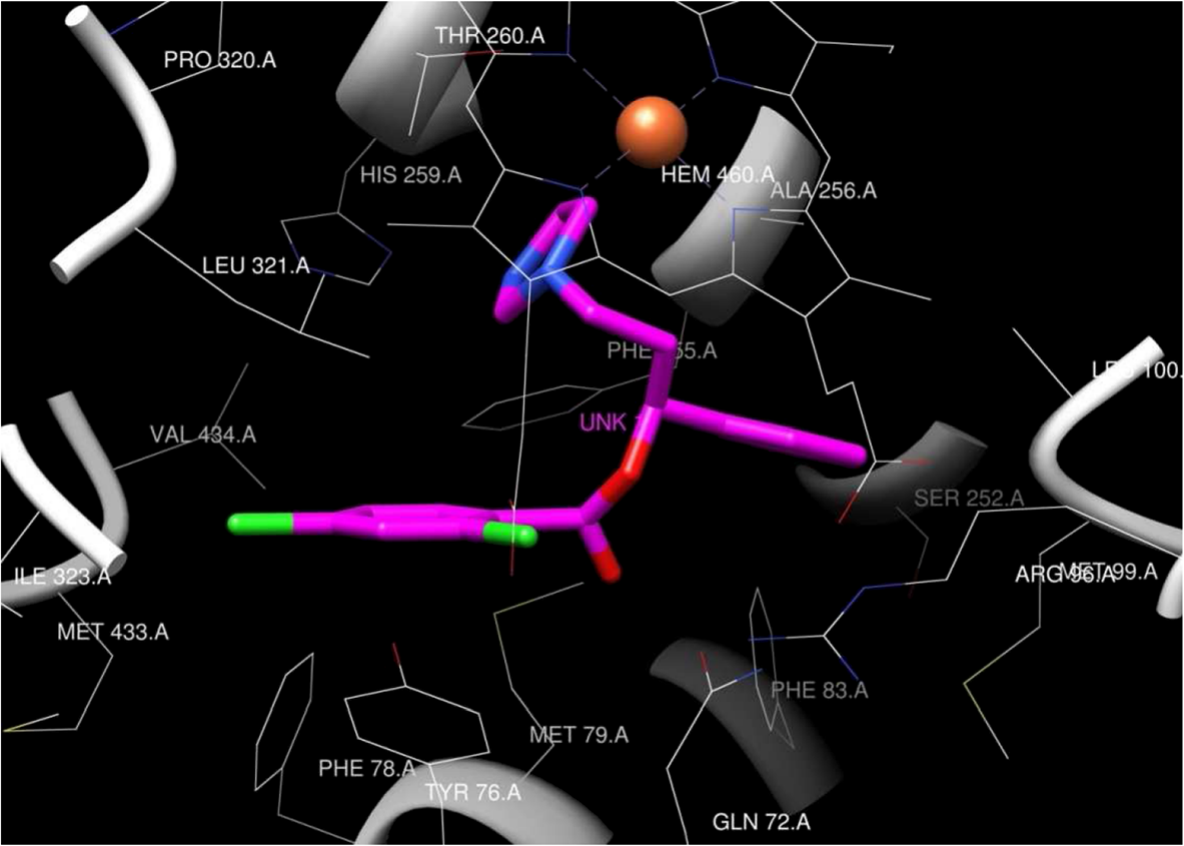
Compound 8 (coloured magenta) docked into 1AE1.

The binding site includes a hydrophobic pocket delineated by the side chains of Gln 72, Tyr 76, Phe 78, Met 79, Phe 83, Arg 96, Met 99, Leu 100, Ser 252, Phe 255, Ala 256, His 259, Leu 321, Ile 323, Met 433 and Val 434 (Figure 9). The docking pose of compound **8** as an example of the designed compounds showed that the imidazole scaffold of compound **8** is oriented in the same binding site as that of the triazole moiety of the co-crystallized FCZ with 1EA1 (Figure 9). The imidazole ring in compound **8** is oriented near the heme residue in the binding site delineated by the Ala 256, His 259, Thr 260 while the phenyl ring of the benzoate moiety is sandwiched between the phenyl ring of Tyr 76 and side chain of Leu 321.

## Conclusion

Synthesis, characterization and anti-*Candida* activity of certain new aryl/heterocyclic esters **5a-k** of 1-aryl-3-(1*H*-imidazol-1-yl) propan-1-ols **4a-d** have been successfully achieved. The synthesized esters **5a-k** exhibited anti-*Candida* activity better than that of the gold standard antifungal drug, fluconazole. Compound **5a** emerged as the most active congener (MIC = 0.0833 μmol/mL) among the newly synthesized compounds **5a-k** being nearly 20 times more potent than fluconazole (MIC >1.6325 μmol/mL) as an anti-*Candida albicans* agent. Compound **5a** showed a slightly weaker activity against *Candida albicans* than that of our previously reported compounds **7** and **8** (MIC = 0.0117 and 0.0104 μmol/mL, respectively). Molecular modeling studies on a set of anti-*Candida albicans* compounds were able to effectively satisfy the proposed pharmacophore geometry.

## Competing interests

The authors declare that they have no competing interests.

## Authors’ contributions

MIA has formulated the research idea, result’s interpretation and discussion and wrote the manuscript. AAR undertook the molecular modeling studies, interpretation of the results and shared in preparation of the manuscript. ASZ carried out anti-*Candida* assays and shared in preparation of the manuscript. MSA assisted with interpretation of the data and participated in preparation of the manuscript. SWG prepared the title compounds and participated in preparation of the manuscript. All authors have read and approved the final manuscript.

## Supplementary Material

Additional file 1 ^**1**^**H and **^**13**^**C NMR spectra of compounds 5c, 5e, 5f, 5g-i and 5k.** Click here for file
