# Supplementary material for: 1-Aryl-3-(1H-imidazol-1-yl)propan-1-ol esters: synthesis, anti-Candida potential and molecular modeling studies
Source: Chem Cent J. 2013 Oct 25;7:168. doi: 10.1186/1752-153X-7-168 (PMC3819650; doi:10.1186/1752-153X-7-168)
Supplement: Additional file 1 — 1 H and 13 C NMR spectra of compounds 5c, 5e, 5f, 5g-i and 5k. [file 1752-153X-7-168-S1.pdf]

-BBO CDCl3 D:\\ π

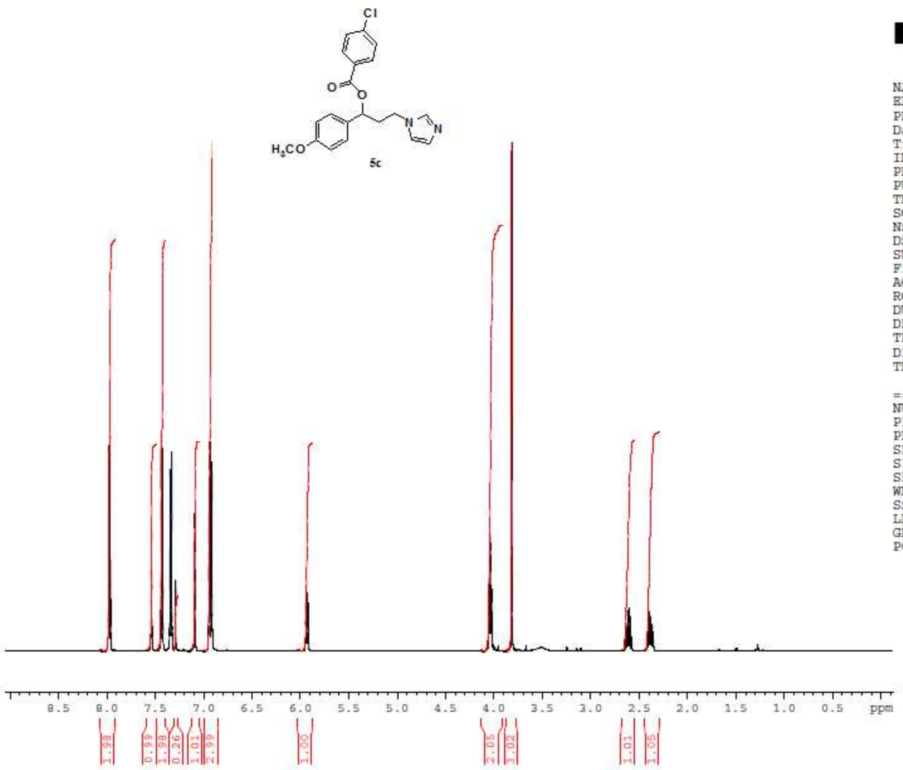

NAME	drattia-SG-27
EXPNO	10
PROCNO	1
Date_	20130113
Time	18.22
INSTRUM	spect
PROBHD	5 mm PABBO BB-
PULPROG	zg30
TD	65536
SOLVENT	CDCl3
NS	16
DS	2
SWH	10330.578 Hz
FIDRES	0.157632 Hz
AQ	3.1720407 sec
RG	90.5
DW	48.400 usec
DE	6.50 usec
TE	297.5 K
D1	1.00000000 sec
TD0	1

======== CHANNEL f1 ========

NUC1	1H
P1	14.70 usec
PL1	-1.00 dB
SFO1	500.1330885 MHz
SI	32768
SF	500.1300000 MHz
WDW	EM
SSB	0
LB	0.30 Hz
GB	0
PC	1.00

BO CDCl3 D:\\ mm

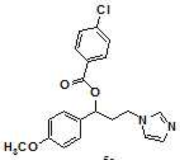

5c

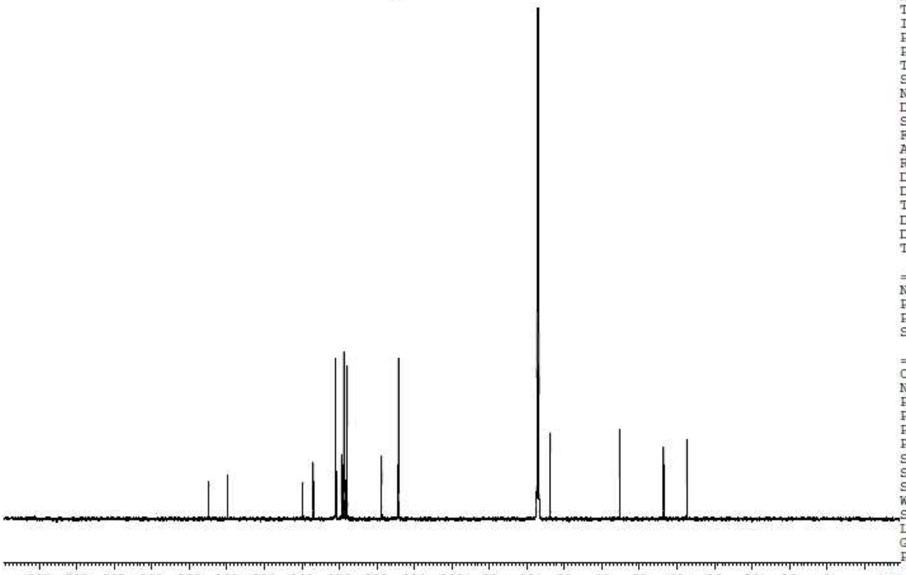

BRUKER

NAME drattia-SG-27
EXPNO 11
PROCNO 1
Date_ 20130113
Time 19.18
INSTRUM spect
PROBHD 5 mm PABBO BB-
PULPROG zgpg30
TD 65536
SOLVENT CDCl3
NS 1024
DS 4
SWH 30030.029 Hz
FIDRES 0.458222 Hz
AQ 1.0912410 sec
RG 11585.2
DW 16.650 usec
DE 6.50 usec
TE 298.3 K
D1 2.00000000 sec
D11 0.03000000 sec
TD0 1

======== CHANNEL f1 ========
NUC1 13C
P1 10.00 usec
PL1 -5.00 dB
SFO1 125.7703643 MHz

======== CHANNEL f2 ========
CPDPRG2 waltz16
NUC2 1H
PCPD2 80.00 usec
PL2 -1.00 dB
PL12 13.40 dB
PL13 16.40 dB
SFO2 500.1320005 MHz
SI 32768
SF 125.7577890 MHz
WDW EM
SSB 0
LB 3.00 Hz
GB 0
PC 1.40

-BBO CDC13 D:\\ m

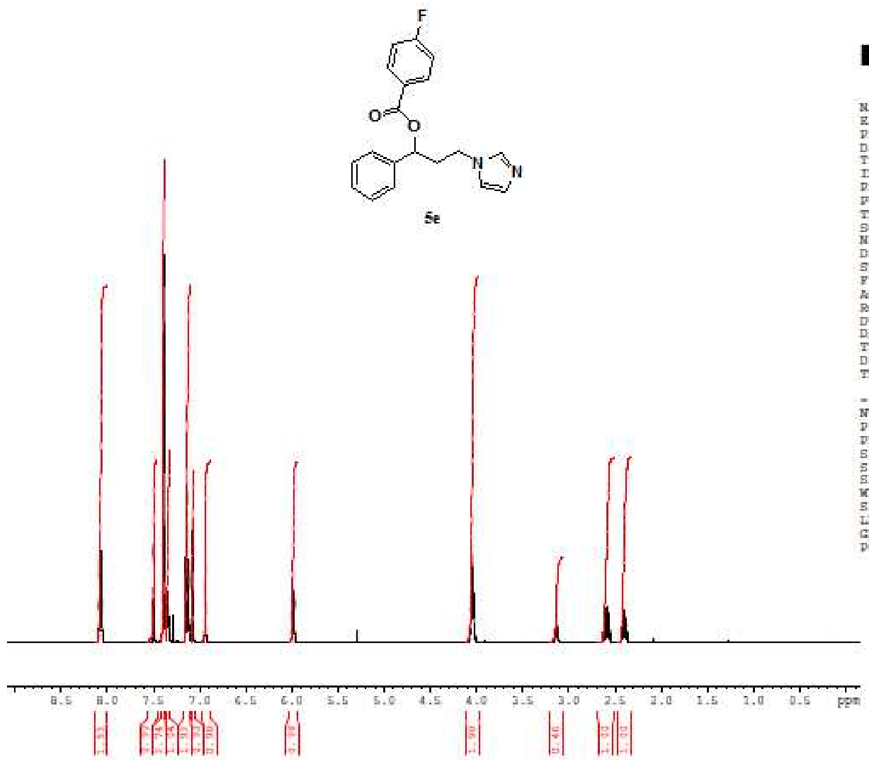

NAME	dråttia-SG-23	
EXPNO	20	
PROCNO	1	
Date_	20121230	
Time	4.42	
INSTRUM	spect	
PROBHD	5 mm PABBO BB-	
PULPROG	zg30	
TD	65536	
SOLVENT	CDC13	
NS	16	
DS	2	
SWH	10330.578	Hz
FIDRES	0.157632	Hz
AQ	3.1720407	sec
RG	35.9	
DW	48.400	usec
DE	6.50	usec
TE	297.5	K
D1	1.00000000	sec
TD0	1	

======== CHANNEL f1 ========

NUC1	1H	
P1	14.70	usec
PL1	-1.00	dB
SFO1	500.1330885	MHz
SI	32768	
SF	500.1300000	MHz
WDW	EM	
SSB	0	
LB	0.30	Hz
GB	0	
PC	1.00	

IBO CDCl3 D:\\ mm

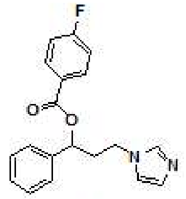

5e

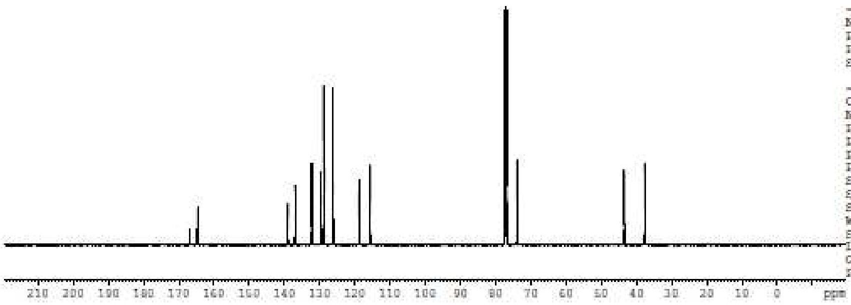

BRUKER

NAME	dratti a-SG-23	
EXPNO	21	
PROCNO	1	
Date_	20121230	
Time	6.32	
INSTRUM	spect	
PROBHD	5 mm PABBO BB-	
PULPROG	zgpg30	
TD	65536	
SOLVENT	CDCl3	
NS	2048	
DS	4	
SWH	30030.029	Hz
FIDRES	0.458222	Hz
AQ	1.0912410	sec
RG	20642.5	
DW	16.650	usec
DE	6.50	usec
TE	298.3	K
D1	2.00000000	sec
D11	0.03000000	sec
TD0	1	

======== CHANNEL f1 ========

NUC1	13C	
P1	10.00	usec
PL1	-5.00	dB
SFO1	125.7703643	MHz

======== CHANNEL f2 ========

CPDPRG2	waltz16	
NUC2	1H	
PCPD2	80.00	usec
PL2	-1.00	dB
PL12	13.40	dB
PL13	16.40	dB
SFO2	500.1320005	MHz
SI	32768	
SF	125.7577890	MHz
WDW	EM	
SSB	0	
LB	3.00	Hz
GB	0	
PC	1.40	

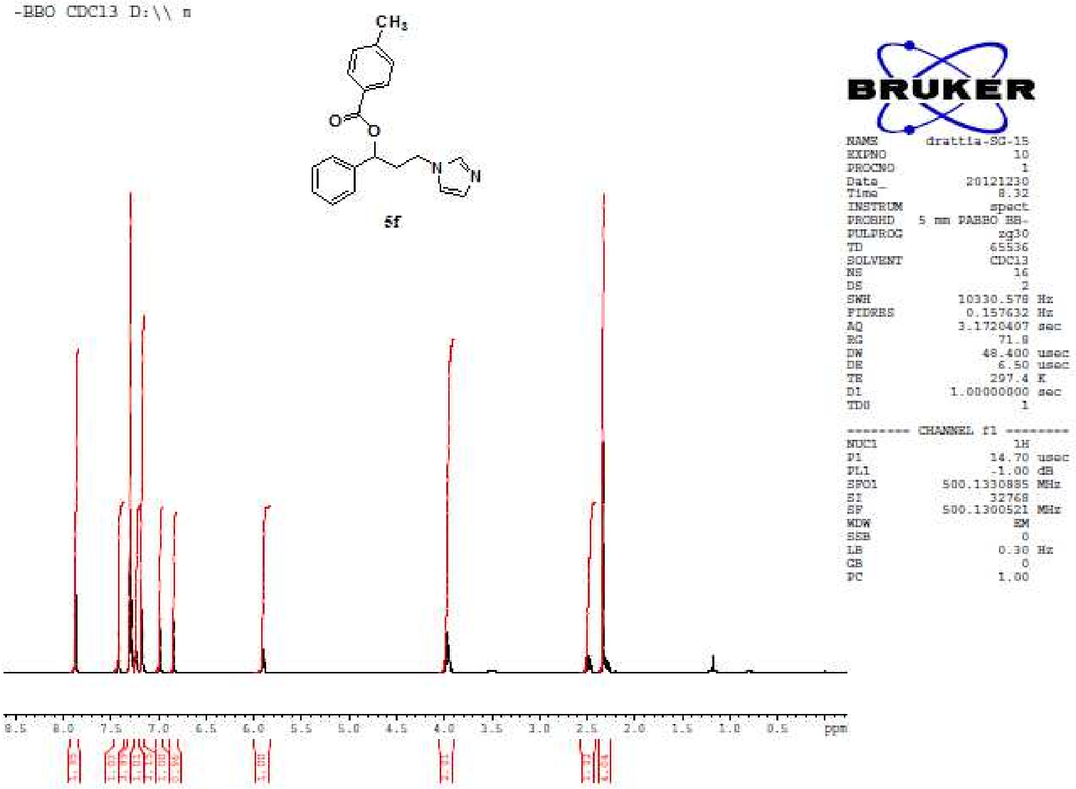
-BBO CDCl3 D:\\ n
CH3
O
O
N
N
5f
BRUKER
NAME drattia-SG-15
EXPNO 10
PROCNO 1
Date_ 20121230
Time 8.32
INSTRUM spect
PROBHD 5 mm PABBO BB-
PULPROG zg30
TD 65536
SOLVENT CDCl3
NS 16
DS 2
SWH 10330.578 Hz
FIDRES 0.157632 Hz
AQ 3.1720407 sec
RG 71.8
DW 48.400 usec
DE 6.50 usec
TE 297.4 K
D1 1.00000000 sec
TD0 1
======== CHANNEL f1 ========
NUC1 1H
P1 14.70 usec
PL1 -1.00 dB
SFO1 500.1330885 MHz
SI 32768
SF 500.1300521 MHz
WDW EM
SSB 0
LB 0.30 Hz
GB 0
PC 1.00
8.5 8.0 7.5 7.0 6.5 6.0 5.5 5.0 4.5 4.0 3.5 3.0 2.5 2.0 1.5 1.0 0.5 ppm

BO CDCl3 D:\\ mm

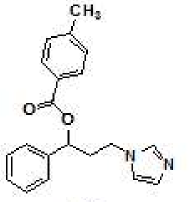

5f

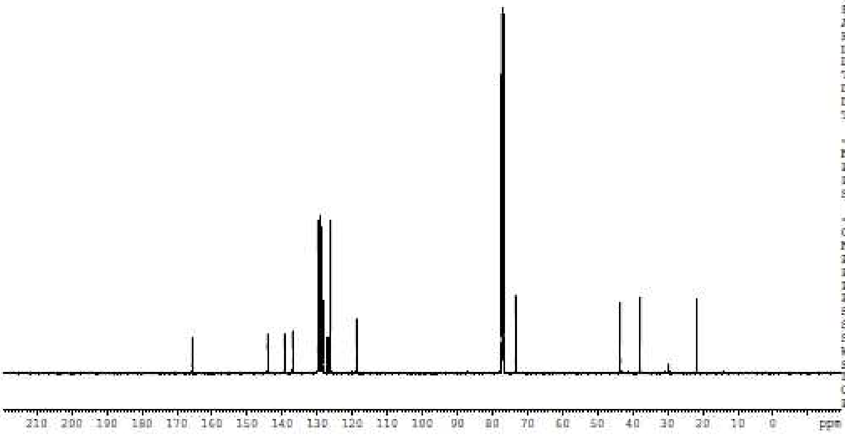


```
NAME        dratt1a-SG-15
EXPNO                  11
PROCNO                  1
Date_            20121230
Time                10.22
INSTRUM             spect
PROBHD   5 mm PABBO BB-
PULPROG           zgpg30
TD                  65536
SOLVENT             CDCl3
NS                   2048
DS                      4
SWH             30030.029 Hz
FIDRES           0.458222 Hz
AQ              1.0912410 sec
RG                  16384
DW                 16.650 usec
DE                   6.50 usec
TE                  298.5 K
D1             2.00000000 sec
D11            0.03000000 sec
TD0                     1

======== CHANNEL f1 ========
NUC1                  13C
P1                  10.00 usec
PL1                 -5.00 dB
SFO1          125.7703643 MHz

======== CHANNEL f2 ========
CPDPRG2           waltz16
NUC2                   1H
PCPD2               80.00 usec
PL2                 -1.00 dB
PL12                13.40 dB
PL13                16.40 dB
SFO2          500.1320005 MHz
SI                  32768
SF            125.7577890 MHz
WDW                    EM
SSB                     0
LB                   3.00 Hz
GB                      0
PC                   1.40
```

-BBO CDCl3 D:\\ m

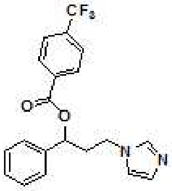

5g

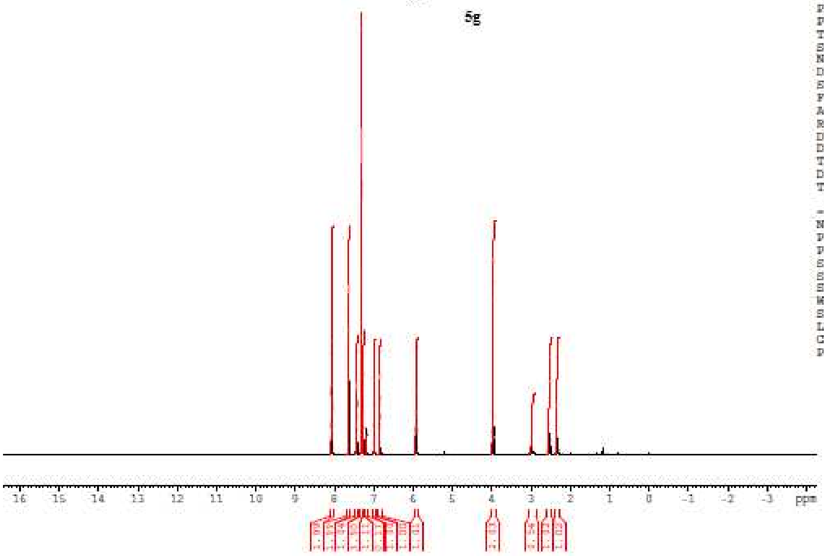

NAME	dratti-SG-22	
EXPNO	20	
PROCNO	1	
Date_	20121230	
Time	6.37	
INSTRUM	spect	
PROBHD	5 mm PABBO BB-	
PULPROG	zg30	
TD	65536	
SOLVENT	CDCl3	
NS	16	
DS	2	
SWH	10330.578	Hz
FIDRES	0.157632	Hz
AQ	3.1720407	sec
RG	71.8	
DW	48.400	usec
DE	6.50	usec
TE	297.4	K
D1	1.00000000	sec
TD0	1	

======== CHANNEL f1 ========

NUC1	1H	
P1	14.70	usec
PL1	-1.00	dB
SFO1	500.1330885	MHz
SI	32768	
SF	500.1300501	MHz
WDW	EM	
SSB	0	
LB	0.30	Hz
GB	0	
PC	1.00	

BO CDC13 D:\\ mm

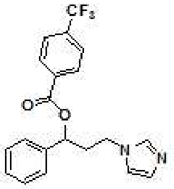

5g

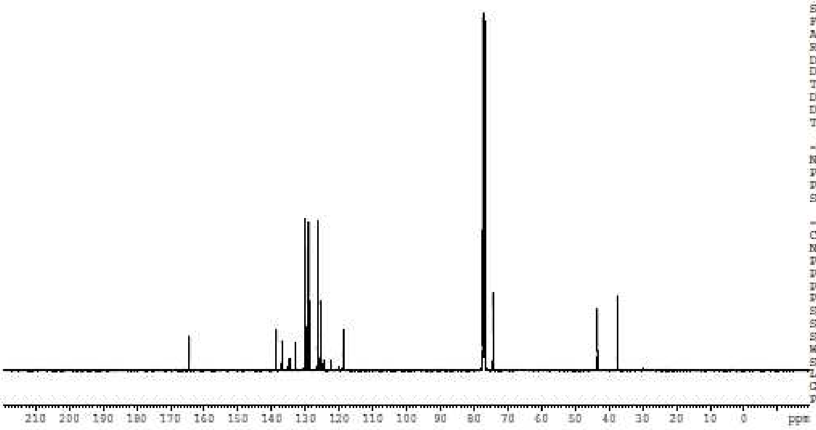

NAME	dratt1a-SG-22	
EXPNO	21	
PROCNO	1	
Date_	20121230	
Time	8.27	
INSTRUM	spect	
PROBHD	5 mm PABBO BB-	
PULPROG	zgpg30	
TD	65536	
SOLVENT	CDC13	
NS	2048	
DS	4	
SWH	30030.029	Hz
FIDRES	0.458222	Hz
AQ	1.0912410	sec
RG	18390.4	
DW	16.650	usec
DE	6.50	usec
TE	298.3	K
D1	2.00000000	sec
D11	0.03000000	sec
TD0	1	
======== CHANNEL f1 ========		
NUC1	13C	
P1	10.00	usec
PL1	-5.00	dB
SFO1	125.7703643	MHz
======== CHANNEL f2 ========		
CPDPRG2	waltz16	
NUC2	1H	
PCPD2	80.00	usec
PL2	-1.00	dB
PL12	13.40	dB
PL13	16.40	dB
SFO2	500.1320005	MHz
SI	32768	
SF	125.7577890	MHz
WDW	EM	
SSB	0	
LB	3.00	Hz
GB	0	
PC	1.40	

-BBO CDCl3 D:\\ m

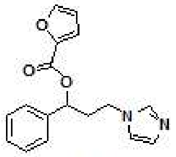

5h

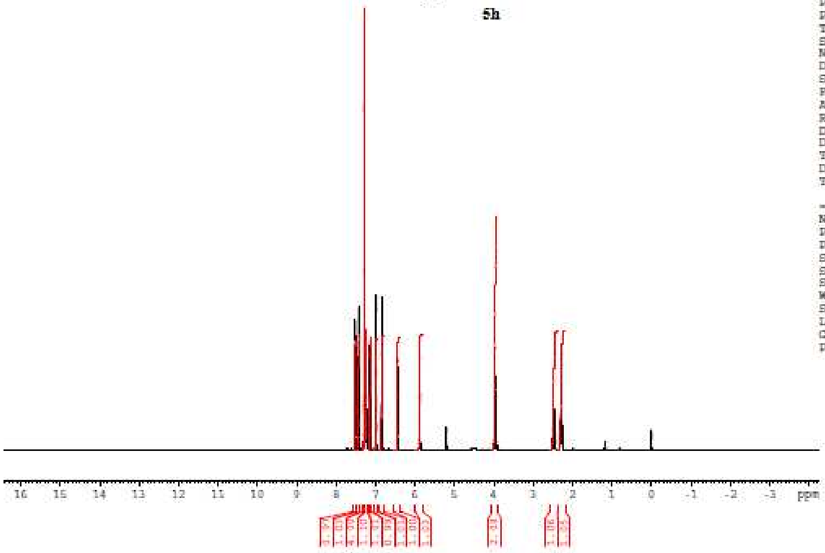

BRUKER

```
NAME          drattia-SG-14
EXPNO                    10
PROCNO                    1
Date_              20121230
Time                  10.27
INSTRUM               spect
PROBHD   5 mm PABBO BB-
PULPROG                zg30
TD                    65536
SOLVENT               CDCl3
NS                       16
DS                        2
SWH           10330.578 Hz
FIDRES         0.157632 Hz
AQ            3.1720407 sec
RG                     71.8
DW             48.400 usec
DE               6.50 usec
TE                297.7 K
D1         1.00000000 sec
TD0                       1

======== CHANNEL f1 ========
NUC1                     1H
P1              14.70 usec
PL1               -1.00 dB
SFO1      500.1330885 MHz
SI                    32768
SF        500.1300453 MHz
WDW                      EM
SSB                       0
LB                  0.30 Hz
GB                        0
PC                     1.00
```

BO CDCl3 D:\\ mm

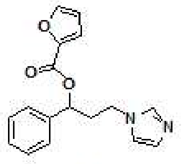

5h

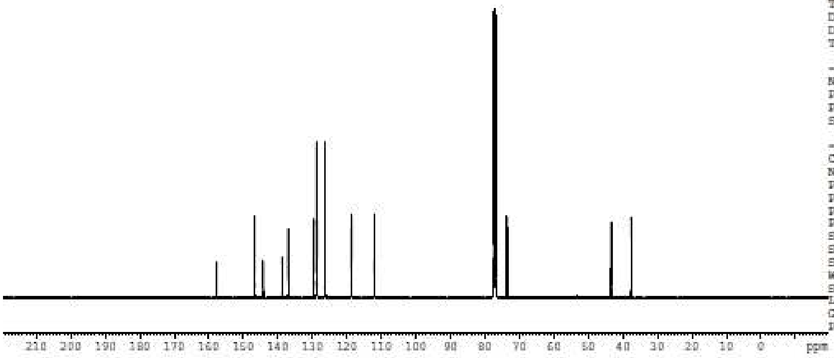

NAME	drattia-SG-14
EXPNO	11
PROCNO	1
Date_	20121230
Time	12.17
INSTRUM	spect
PROBHD	5 mm PABBO BB-
PULPROG	zgpg30
TD	65536
SOLVENT	CDCl3
NS	2048
DS	4
SWH	30030.029 Hz
FIDRES	0.458222 Hz
AQ	1.0912410 sec
RG	20642.5
DW	16.650 usec
DE	6.50 usec
TE	298.6 K
D1	2.00000000 sec
D11	0.03000000 sec
TD0	1
======== CHANNEL f1 ========	
NUC1	13C
P1	10.00 usec
PL1	-5.00 dB
SFO1	125.7703643 MHz
======== CHANNEL f2 ========	
CPDPRG2	waltz16
NUC2	1H
PCPD2	80.00 usec
PL2	-1.00 dB
PL12	13.40 dB
PL13	16.40 dB
SFO2	500.1320005 MHz
SI	32768
SF	125.7577890 MHz
WDW	EM
SSB	0
LB	3.00 Hz
GB	0
PC	1.40

-BBO CDCl3 D:\\ π

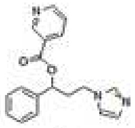

[illegible]

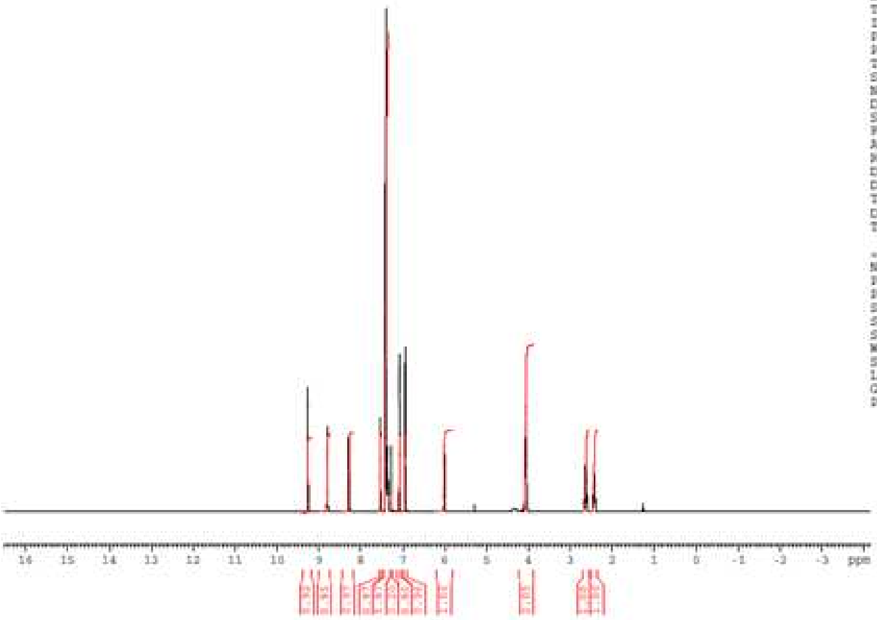

NAME dhattia-SG-12
EXPNO 10
PROCNO 1
Date_ 20121211
Time 14.47
INSTRUM spect
PROBHD 5 mm PABBO BB-
PULPROG zg30
TD 65536
SOLVENT CDCl3
NS 16
DS 2
SWH 10330.578 Hz
FIDRES 0.157632 Hz
AQ 3.1720407 sec
RG 90.5
DW 48.400 usec
DE 6.50 usec
TE 299.9 K
D1 1.00000000 sec
TD0 1

======== CHANNEL f1 ========
NUC1 1H
P1 14.70 usec
PL1 -1.00 dB
SFO1 500.1330885 MHz
SI 32768
SF 500.1300000 MHz
WDW EM
SSB 0
LB 0.30 Hz
GB 0
PC 1.00

BO CDCl3 D:\\ mm

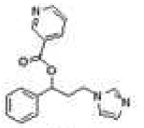

5i

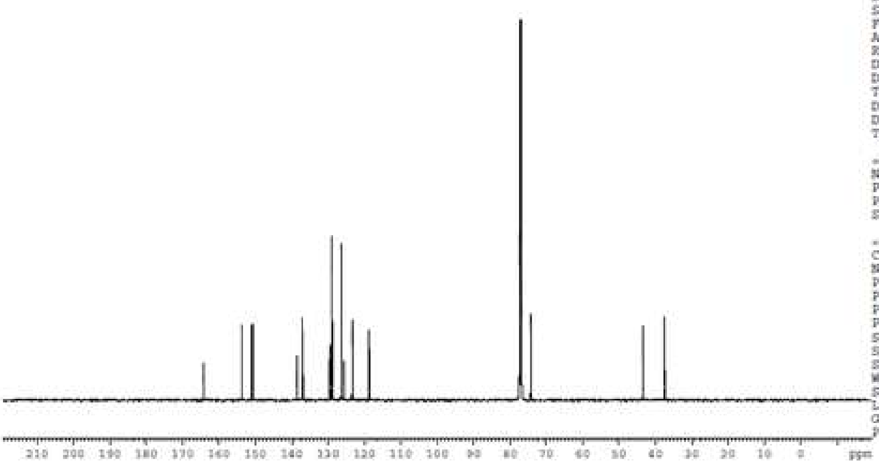

NAME	drattia-SG-12	
EXPNO	11	
PROCNO	1	
Date_	20121211	
Time	16.37	
INSTRUM	spect	
PROBHD	5 mm PABBO BB-	
PULPROG	zgpg30	
TD	65536	
SOLVENT	CDCl3	
NS	2048	
DS	4	
SWH	30030.029	Hz
FIDRES	0.458222	Hz
AQ	1.0912410	sec
RG	18390.4	
DW	16.650	usec
DE	6.50	usec
TE	300.9	K
D1	2.00000000	sec
D11	0.03000000	sec
TD0	1	

======== CHANNEL f1 ========

NUC1	13C	
P1	10.00	usec
PL1	-5.00	dB
SFO1	125.7703643	MHz

======== CHANNEL f2 ========

CPDPRG2	waltz16	
NUC2	1H	
PCPD2	80.00	usec
PL2	-1.00	dB
PL12	13.40	dB
PL13	16.40	dB
SFO2	500.1320005	MHz
SI	32768	
SF	125.7577890	MHz
WDW	EM	
SSB	0	
LB	3.00	Hz
GB	0	
PC	1.40	

-BBO CDCl3 D:\\ n

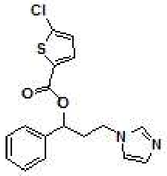

5k

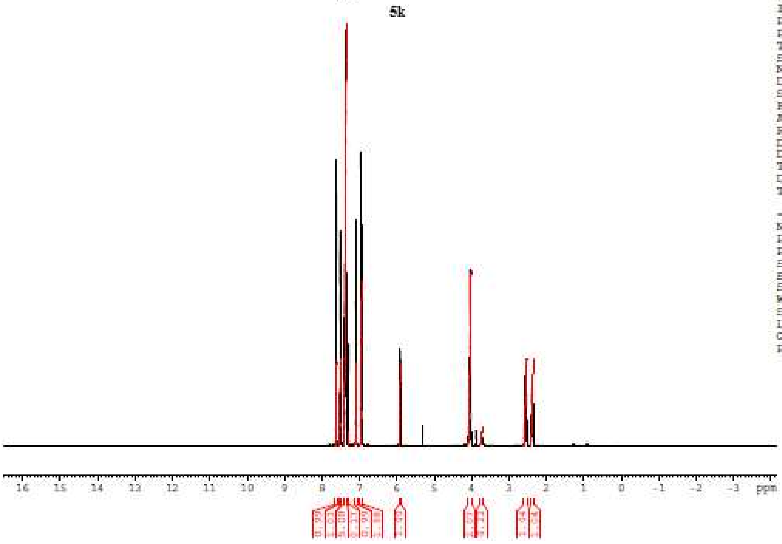

NAME	drattia-SG-24	
EXPNO	10	
PROCNO	1	
Date_	20121126	
Time	6.27	
INSTRUM	spect	
PROBHD	5 mm PABBO BB-	
PULPROG	zg30	
TD	65536	
SOLVENT	CDCl3	
NS	16	
DS	2	
SWH	10330.578	Hz
FIDRES	0.157632	Hz
AQ	3.1720407	sec
RG	90.5	
DW	48.400	usec
DE	6.50	usec
TE	298.0	K
D1	1.00000000	sec
TD0	1	

======== CHANNEL f1 ========

NUC1	1H	
P1	14.70	usec
PL1	-1.00	dB
SFO1	500.1330885	MHz
SI	32768	
SF	500.1300000	MHz
WDW	EM	
SSB	0	
LB	0.30	Hz
GB	0	
PC	1.00	

!BO CDCl3 D:\\ mm

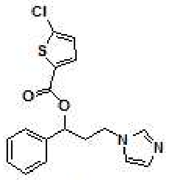

5k

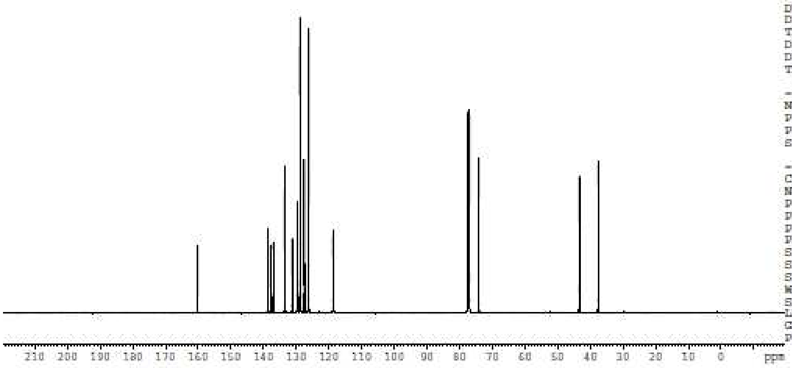

NAME dratti a-SG-24
EXPNO 21
PROCNO 1
Date_ 20121230
Time 4.37
INSTRUM spect
PROBHD 5 mm PABBO BB-
PULPROG zgpg30
TD 65536
SOLVENT CDCl3
NS 2048
DS 4
SWH 30030.029 Hz
FIDRES 0.458222 Hz
AQ 1.0912410 sec
RG 20642.5
DW 16.650 usec
DE 6.50 usec
TE 298.3 K
D1 2.00000000 sec
D11 0.03000000 sec
TD0 1

======== CHANNEL f1 ========
NUC1 13C
P1 10.00 usec
PL1 -5.00 dB
SFO1 125.7703643 MHz

======== CHANNEL f2 ========
CPDPRG2 waltz16
NUC2 1H
PCPD2 80.00 usec
PL2 -1.00 dB
PL12 13.40 dB
PL13 16.40 dB
SFO2 500.1320005 MHz
SI 32768
SF 125.7577890 MHz
WDW EM
SSB 0
LB 3.00 Hz
GB 0
PC 1.40
